# Antiallergic Effects of N,N-dicoumaroylspermidine Isolated from *Lithospermum erythrorhizon* on Mast Cells and Ovalbumin-Induced Allergic Rhinitis

**DOI:** 10.3390/ijms231810403

**Published:** 2022-09-08

**Authors:** Tam Thi Le, Tae Kyeom Kang, Wook-Bin Lee, Sang Hoon Jung

**Affiliations:** 1 Natural Product Research Center, Korea Institute of Science & Technology, Gangneung 25451, Korea; 2 Division of Bio-Medical Science & Technology, KIST School, Korea University of Science and Technology, Gangneung 25451, Korea

**Keywords:** *Lithospermum erythrorhizon*, allergic rhinitis, N1″,N3″-dicoumaroylspermidine, erythrin A, erythrin B, antiallergic

## Abstract

In East Asia, the dried root of *Lithospermum erythrorhizon* has been utilized as an anti-inflammatory, antipyretic, detoxifying, and anti-inflammatory agent. Recently, we reported that *L. erythrorhizon* protects against allergic rhinitis; however, the component within *L. erythrorhizon* that exerts antiallergic activity remains unknown. The purpose of the current study was to isolate and characterize the antiallergic active components in an ethanolic extract of *L. erythrorhizon* roots. We examined the antiallergic effects of *L. erythrorhizon* reflux ethanol extracts in an ovalbumin (OVA)-induced allergic rhinitis mouse model, and compared the chemical compounds extracted using the hot reflux and cold extraction methods. Chromatographic separation identified two novel anthraquinones, erythrin A and B, one newly discovered compound from the *Lithospermum* genus, N1″,N3″-dicoumaroylspermidine, and nineteen other recognized compounds. Their chemical structures were elucidated by single (1D) and 2D analysis of nuclear magnetic resonance (NMR) spectroscopic data, as well as high resolution mass spectrometry. Among the identified compounds, N,N′-dicoumaroylspermidine strongly inhibited the release of β-hexosaminidase, as well as the production of IL-3, IL-4, and IL-13 by IgE-sensitized and BSA-stimulated RBL-2H3 cells. Using the OVA-induced allergic rhinitis mouse model, we showed that N,N′-dicoumaroylspermidine reduced the production of serum OVA-specific IgE and the number of inflammatory cells in nasal lavage fluid. N,N′-dicoumaroylspermidine isolated from *L. erythrorhizon* exhibits antiallergic properties, making it potentially effective for allergic rhinitis.

## 1. Introduction

Allergic rhinitis is an inflammatory disease of the nasal passages triggered by allergen exposure. It is estimated that over 400 million people worldwide have allergic rhinitis [1]. Mold, mites, and pet hair, all of which are common allergens found in the home, can cause allergic rhinitis. Common allergens found outdoors include tree pollen, grass, and mold spores [2]. Allergic rhinitis is a clinical term that refers to a condition in which symptoms such as sneezing and a stuffy, itchy, and runny nose occur soon after exposure to an allergen. If the symptoms are left untreated, they can have serious health consequences such as insomnia, drowsiness, and depression, which reduce an individual’s quality of life [3] and productivity, often incurring significant economic costs [4]. Antihistamines, steroids, and immunosuppressants are used to alleviate the symptoms of allergic rhinitis [5]. Chronic use of such medications, however, often results in unfavorable side effects [6,7]. Thus, it is important to discover new therapeutic agents derived from medicinal plants, which in general have fewer side effects and are safe to use.

Mast cells and basophils are the main cell types responsible for allergic rhinitis [8]. Mast cells express the immunoglobulin Fc epsilon receptor I (FcεRI), a high-affinity IgE receptor that mediates acute and chronic inflammation in response to IgE-mediated activation via the production of allergic mediators. Following the engagement of antigen-binding IgE antibodies with surface membrane receptors, mast cells secrete histamines, prostaglandins, leukotrienes, and cytokines. These cytokines promote neutrophil and macrophage chemotaxis and phagocytosis. Additionally, the cytokine-mediated reaction results in tissue inflammation; therefore, limiting the activity of mast cells and basophils is necessary to treat allergic inflammation [9].

*L. erythrorhizon* is common in East Asian countries such as Korea, China, and Japan [10]. The roots of *L. erythrorhizon* have been used for many purposes, such as healing burns and wounds, as an ingredient in anti-inflammation ointments [11], as a dye for staining fabrics, and as a natural food colorant. The red pigment derived from the roots of *L. erythrorhizon* comprises shikonin and its ester derivatives [12]. These components are responsible for a wide range of anticancer, antifungal, and antioxidant effects [13,14]. Additionally, an animal model of ocular injury revealed that the antioxidant effects of *L. erythrorhizon* can enhance retinal cell death by reducing the loss of optic nerve axons, and by protecting optic nerve projections and visual function; in addition, lithospermic acid A, isolated from *L. erythrorhizon*, may protect against retinal cell death induced by excitotoxicity and oxidative stress [10]. Our own research recently demonstrated the antiallergic rhinitis effects of *L. erythrorhizon* [15]. However, we still do not know which component within *L. erythrorhizon* exerts the antiallergic effects. Here, we aimed to further investigate the chemical components of *L. erythrorhizon* extracts to identify those that mediate antiallergic rhinitis properties.

## 2. Results

### 2.1. HPLC Analysis of Extracts of L. Erythrorhizon

**Figure 1:**
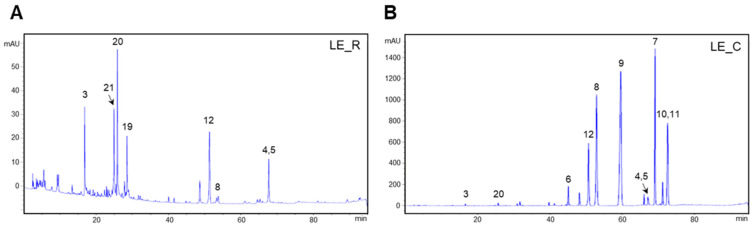
HPLC chromatogram of *L. erythrorhizon* extracts derived from the cold and heat extraction procedures. (**A**) LE_R, 95% ethanol reflux extraction at 70 °C. (**B**) LE_C, 95% ethanol extraction at room temperature. (3) N,N′-dicoumaroylspermidine, (4) anhydroalkannin, (5) deoxyshikonin, (6) shikonin, (7) isobutyrylshikonin, (8) β-hydroxyisovalerylshikonin, (9) acetylshikonin, (10) α-methylbutyrylshikonin, (11) isovalerylshikonin, (12) shikonofuran E, (19) salvianolic acid A, (20) lithospermic acid, and (21) rosmarinic acid.

**Figure 2:**
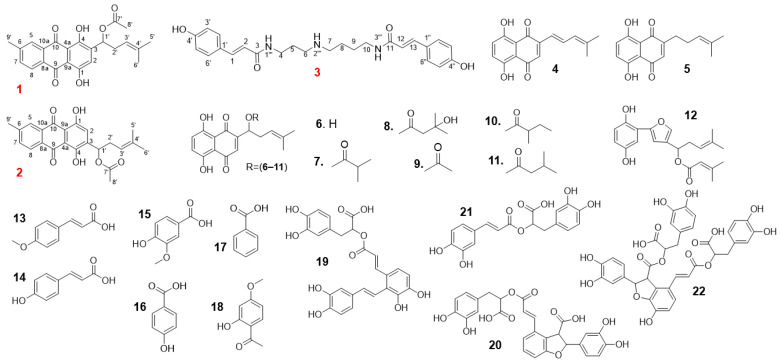
Structures of compounds isolated from *L. erythrorhizon*. Red, two novel compounds **1**, **2**, and one newly discovered compound from the *Lithospermum* genus **3**. Black, known compounds.

We sought to determine which components of the *L. erythrorhizon* reflux extracts (LE_R) have anti-inflammatory or antiallergy properties. Since shikonin in LE_R is thermally unstable, we expected that the composition of the extract would alter considerably after the hot reflux and cold extraction procedures [16,17]. Thus, we used HPLC to compare compounds extracted using these two methods. We detected 12 compounds in LE_R, including the first isolated compound N,N′-dicoumaroylspermidine and two novel compounds (Figure 1A,B). The reflux extract was subjected to repeated column chromatography on silica gel and Sephadex LH-20, resulting in the isolation of two novel compounds, erythrin A (**1**) and erythrin B (**2**), as well as the compound first isolated from *L. erythrorhizon*, N,N′-dicoumaroylspermidine (**3**); we also identified nine other known compounds (**13**–**17** and **19**–**22**) (Figure 2).

Similarly, ten known compounds (**4**–**12**, **18**) were isolated from the cold extract using repeated passage through open columns. All of these isolates were elucidated by comparing their physical and spectroscopic data with those provided in the literature. The known compounds were as follows: anhydroalkannin (**4**) [18], deoxyshikonin (**5**) [19], shikonin (**6**) [12], isobutyrylshikonin (**7**) [12], β-hydroxyisovalerylshikonin (**8**) [12], acetylshikonin (**9**) [12], α-methylbutyrylshikonin (**10**) [12], isovalerylshikonin (**11**) [12], shikonofuran E (**12**) [20], *p*-methoxycinnamic acid (**13**) [21], *p*-coumaric acid (**14**) [22], vanillic acid (**15**) [22], 4-hydroxybenzoic acid (**16**) [22], benzoic acid (**17**) [22], paeonol (**18**) [23], salvianolic acid A (**19**) [24], lithospermic acid (**20**) [25], rosmarinic acid (**21**) [25], and lithospermic acid B (**22**) [25] (Figure 2).

**Figure 3:**
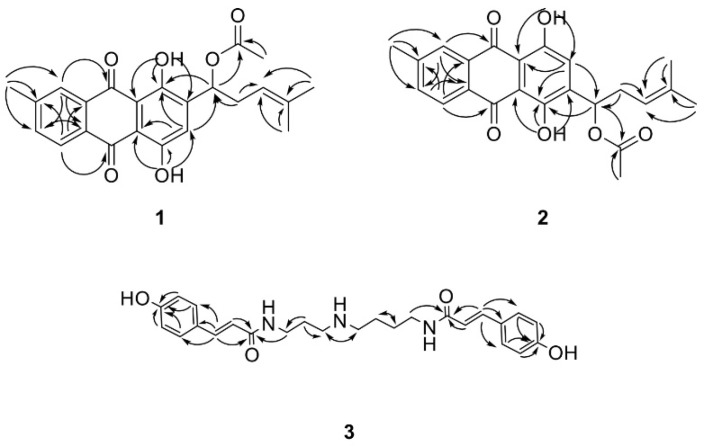
Key HBMC correlations exhibited by compounds **1**, **2**, and **3**.

A mixture of compounds **1** and **2** was obtained as an orange gum with a molecular formula of C_36_H_30_O_16_ from the ion peak at *m*/*z* 393.1339 [M-H]^−^ (calcd. for C_36_H_29_O_16_, 393.1338) in the negative-ion HR-ESIMS (Figure S1). The ^1^H and ^13^C NMR spectra of the mixture revealed duplicate signals, indicating a close structural similarity between compounds **1** and **2**, suggesting that they likely comprise a pair of isomers with the molecular formula C_36_H_30_O_16_ (Figures S2 and S3, Table 1). The ^1^H spectra showed two phenolic hydroxyl signals (δ 12.98 and 13.39) for compound **1** and two for compound **2** (δ 12.94 and 13.43), two sets of 1,3,4-trisubstituted benzene ring signals [2 d, J = 7.96), 7.65 (1 H, m)] for 1, [δ 8.16 (1 H, m), 8.24 (1 H, d, J = 7.96), 7.65 (1 H, m)] for 2], two singlets due to an aromatic proton at δ 7.31 for **1** and 7.30 for **2**, one double bond at δ 5.17, and four methyl groups at δ 1.59, 1.71, 2.16, and 2.56 for both **1** and **2**. The ^13^C NMR spectra revealed the presence of one carboxyl group (δ 170.10) and two ketone carbonyl groups [(δ 186.63 and 187.18) in **1** and δ 186.97 and 187.33 in **2**]. CH_3_ 5′, 6′, 8′, and 9′ were located by the correlation from H-5′, 6′ to C-4′, 3′, from H-8′ to C-7′, and from H-9′ to C-5,6 (Figure 3). Taking all these spectroscopic data into account, compounds **1** and **2** were identified as 1,4-dihydroxy-3-(1′-acetoxy-4′-methylpent-3′-enyl)-9′-methylanthracene-9,10-dione and 1,4-dihydroxy-2-(1′-acetoxy-4′-methylpent-3′-enyl)-9′-methylanthracene-9,10-dione, respectively, and named erythrin A and erythrin B, respectively.

Compound **3** was a colorless gum with a molecular formula of C_36_H_30_O_16_ from the ion peak at *m*/*z* 438.2387 [M+H]^+^ (calcd. for C_25_H_32_N_3_O_4,_ 438.2393) in the positive-ion HR-ESIMS (Figure S7). The ^1^H spectra showed two sets of A2B2 spin systems [δ 6.80 (2 H, d, *J* = 8.56), 7.41 (2 H, d, *J* = 8.64)) and (δ 6.80 (2 H, d, *J* = 8.56), 7.42 (2 H, d, *J* = 8.68)], two double bond signals [δ 6.42 (1 H, d, *J* = 15.68), 7.51 (1 H, d, *J* = 15.56) and 6.42 (1 H, d, *J* = 15.68), 7.47 (1 H, d, *J* = 15.56)], and seven methylenes [δ 3.37 (2 H, t, *J* = 6.72), 3.43 (2 H, t, *J* = 6.40), 1.65–3.08 (m)] (Figure S8, Table 2). The ^13^C NMR spectra indicated two carbamoyl groups (δ 168.05 and 168.93) (Figure S9, Table 2). The correlation from H-4 to C-3 and H-10 to C-11 confirmed the position of two coumaroyl groups (Figure 3). A previous study that compared the retention time and ESI-MS of N,N′-dicoumaroylspermidine from the *Aphelandra* species with that of synthetic reference compounds [26] was the first to isolate compound **3** from the *Lithospermum* genus.

### 2.2. Effects of L. erythrorhizon Reflux Ethanol Extract on Levels of Th2 Cytokines in Serum and the Nasal Lavage Fluid of OVA-Induced Allergic Rhinitis Mice

**Figure 4:**
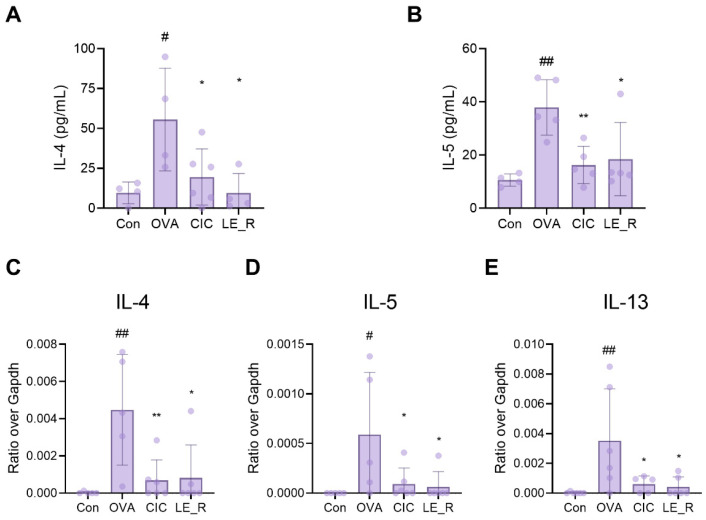
Effect of *L. erythrorhizon* reflux ethanol extract on cytokine levels in the serum and nasal lavage fluid of mice with OVA-induced allergic rhinitis. The concentrations of (**A**) IL-4 and (**B**) IL-5 in the serum of mice were measured using ELISA kits. Levels of (**C**) IL-4, (**D**) IL-5, and (**E**) IL-13 mRNA in NALF were measured by quantitative real-time RT-PCR. Data are expressed as the mean ± SD (*n* = 4~6 per group). # *p* < 0.05, ## *p* < 0.01 versus the Control group. * *p* < 0.05, ** *p* < 0.01 versus the ovalabumin group. Con, control; OVA, ovalbumin; CIC, ciclesonide; LE_R, reflux ethanol extract of *L. erythrorhizon*.

To investigate whether LE_R alleviates allergic reactions in an OVA-induced allergic rhinitis mice model, we used ELISA kits to measure the amounts of the T-helper (Th) type 2 cytokines IL-4 and IL-5 in the serum. Compared with the control group, OVA-induced allergic rhinitis mice showed higher levels of both these Th2-related cytokines. However, these levels fell significantly in the presence of ciclesonide (CIC; used as a positive control) or LE_R (Figure 4A,B). Next, we examined the expression of mRNA encoding Th2-related cytokines in the nasal lavage fluid (NALF) of OVA-induced allergic rhinitis mice. The expression of IL-4, IL-5, and IL-13 mRNA was significantly higher in mice with OVA-induced allergic rhinitis than in the control group; however, they were significantly lower in mice treated additionally with LE_R or CIC (Figure 4C–E).

### 2.3. L. erythrorhizon Reflux Ethanol Extract Causes Histopathological Changes in Tissues from OVA-Induced Allergic Rhinitis Mice Model

**Figure 5:**
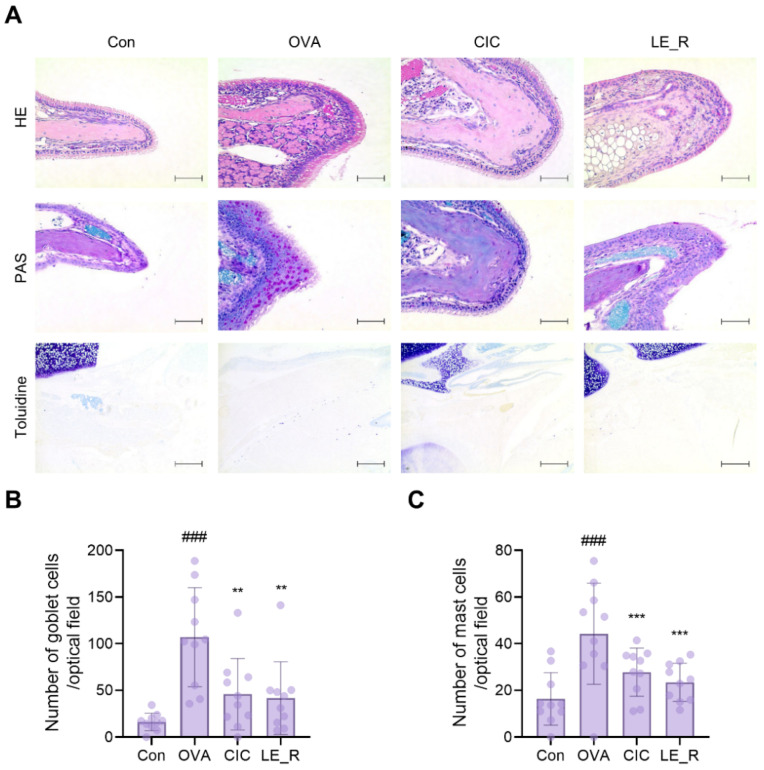
The *L. erythrorhizon* reflux ethanol extract causes histological changes in the nasal tissues of mice with OVA-induced allergic rhinitis. (**A**) Hematoxylin and eosin (HE), Periodic Acid-Schiff (PAS), and toluidine blue staining of nasal tissue samples from each group (scale bars, 400 μm). Quantification of staining in histological sections enables counting of (**B**) goblet cells (PAS staining) and (**C**) mast cells (toluidine blue staining). Data are expressed as the mean ± SD (*n* = 10 per group). Significant differences at ### *p* < 0.001 versus the Control group; ** *p* < 0.01, *** *p* < 0.001 versus the ovalbumin group. Con, control; OVA, ovalbumin; CIC, ciclesonide; LE_R, reflux ethanol extract of *L. erythrorhizon*.

Head tissue sections were stained with H&E, PAS, and toluidine blue to examine the protective effects of *L. erythrorhizon*. H&E staining revealed epithelial disruption and mucosal detachment in OVA-treated allergic rhinitis mice, while mice treated with CIC or LE_R were protected from OVA-induced damage (Figure 5A). Additionally, PAS staining revealed a significant increase in goblet cell hyperplasia in the OVA group, which was suppressed significantly by CIC or LE_R (Figure 5A,B). The number of mast cells in the nasal mucosa was counted after toluidine blue staining. Mast cells were significantly more abundant in the OVA group than in the control group. By contrast, LE_R significantly reduced mast cell infiltration into the nasal cavity, implying that LE_R inhibits mast cell infiltration into the nasal mucosa (Figure 5C). These results suggest that LE_R may reduce OVA-induced allergic rhinitis symptoms by inhibiting both the release of Th2-related cytokines and inflammatory cell infiltration.

### 2.4. The Effects of 11 Constituents on Viability of THP1 Macrophages and RBL-2H3 Basophils

**Figure 6:**
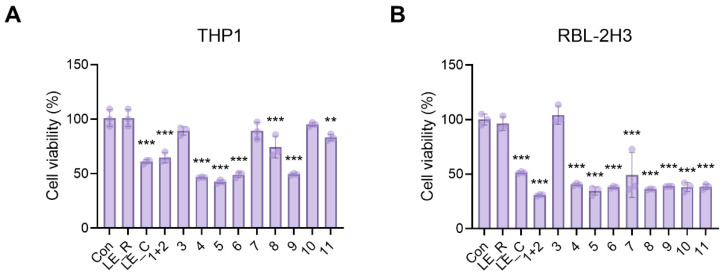
Cytotoxic effects of compounds isolated from the *L. erythrorhizon* cold and reflux EtOH extracts against THP1 and RBL-2H3 cells. (**A**,**B**) Viability of (**A**) THP1 and (**B**) RBL-2H3 cells, as assessed in an MTT assay after 24 h exposure to the indicated extracts (50 μg/mL) or isolated compounds (50 μM). LE_R, 95% ethanol reflux extraction at 70 °C; LE_C, 95% ethanol extraction at room temperature: (1) erythrin A, (2) erythrin B, (3) N,N′-dicoumaroylspermidine, (4) anhydroalkannin, (5) deoxyshikonin, (6) shikonin, (7) isobutyrylshikonin, (8) β-hydroxyisovalerylshikonin, (9) acetylshikonin, (10) α-methylbutyrylshikonin, and (11) isovalerylshikonin. Data are expressed as the mean ± SD. ** *p* < 0.01 and *** *p* < 0.001 versus the control group. Experiments were independently repeated three times.

The human cell line THP1 has been used extensively to study macrophage functions, while the rat cell line RBL-2H3 is an effective in vitro model for mast cells and basophils. Thus, we performed cell viability assays using the 11 components isolated from *L. erythrorhizon*. We have previously demonstrated that LE_R has no discernible effect on cell viability [15]. As shown in Figure 6, LE_R, N,N′-dicoumaroylspermidine (3), α-methylbutyrylshikonin (10), and isovalerylshikonin (11) were not toxic to THP1 cells after 24 h of treatment. Additionally, the LE_R and N,N′-dicoumaroylspermidine (3) were non-toxic to RBL-2H3 cells. Thus, we decided to examine the anti-inflammatory and antiallergic properties of N,N′-dicoumaroylspermidine.

### 2.5. Assessment of NF-kB, AP1, and IRF Activation

**Figure 7:**
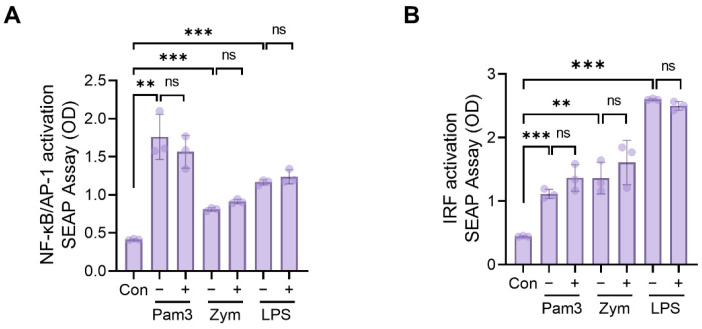
N,N′-dicoumaroylspermidine has no effect on activation of NF-kB, AP-1, and IRF. (**A**) NF-kB, AP-1, and (**B**) IRF reporter cells (THP1-Xblue and THP1-Blue-ISG cells, respectively) were preincubated with (+) or without (−) 50 μM of N,N′-dicoumaroylspermidine for 1 h, followed by stimulation for 24 h with the TLR2/1 agonist Pam3 (100 ng/mL), the TLR2/6 agonist Zym (10 μg/mL), and the TLR4 agonist LPS (100 ng/mL). Data are expressed as the mean ± SD. ** *p* < 0.01 and *** *p* < 0.001 versus the control group. ns = not significant (*t*-test). Experiments were independently repeated three times. Con, control; Pam3, Pam3CSK4; Zym, zymosan A; LPS, lipopolysaccharides.

The NF-κB, AP1, and IRF transcription factor families play important roles in inflammatory responses by promoting the transcription of a variety of cytokines, including TNFα, IL-6, and IFN-α/β [27]. In addition, toll-like receptors (TLRs) activate a common signaling pathway that results in the induction of inflammatory cytokines such as TNFα and IL-6 via the activation of NF-κB, AP1, and IRF. To determine whether N,N′-dicoumaroylspermidine regulates NF-κB, AP1, or IRF activity, we used THP1-XBlue (for NF-κB/AP1 activity) and THP1-ISG-Blue (for IRF activity) reporter cells stimulated with TLR2 or TLR4. These cells release inducible reporter proteins upon the activation of the NF-κB/AP1 and IRF (secreted embryonic alkaline phosphatase) pathways. First, the cells were pretreated for 1 h with or without N,N′-dicoumaroylspermidine and then stimulated for 24 h with a TLR2 ligand (Pam3CSK4 or zymosan) or a TLR4 ligand (lipopolysaccharide (LPS)). Pam3CSK4, zymosan, and LPS all significantly increased NF-κB/AP1 and IRF activity. However, N,N′-dicoumaroylspermidine had no effect on NF-κB/AP1 and IRF activity when treated in addition (Figure 7A,B).

### 2.6. Antiallergic Effects of N,N′-dicoumaroylspermidine on Mast Cells (RBL-2H3)

**Figure 8:**
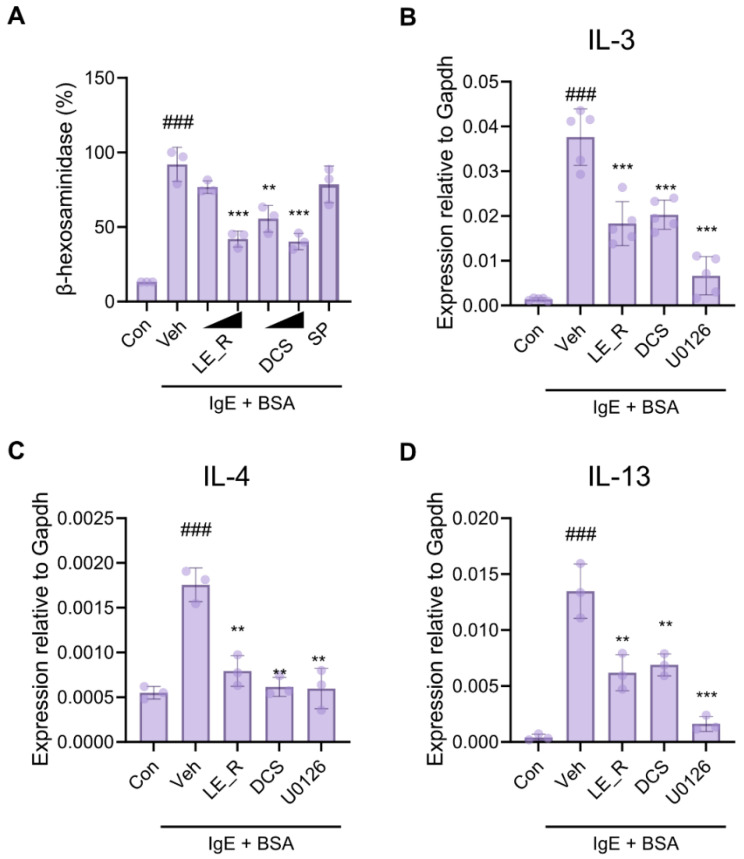
N,N′-dicoumaroylspermidine exhibits antiallergic effects by inhibiting degranulation and cytokine expression by RBL-2H3 cells. FcεRI-mediated release of β-hexosaminidase and cytokine expression. (**A**) RBL-2H3 cells were sensitized with anti-DNP-IgE and treated with LE_R (50 μg/mL), DCS (10, 50 μM), or SP (50 μM) for 1 h, and then challenged with DNP-BSA. Release of β-hexosaminidase was detected after stimulation with DNP-BSA. (**B**–**D**) RBL-2H3 cells were sensitized with anti-DNP-IgE and treated with LE_R (50 μg/mL), DCS (50 μM), or U0126 (10 μM) for 1 h, and then challenged with DNP-BSA. Expression of mRNA encoding IL-3 (**B**), IL-4 (**C**), and IL-13 (**D**) was determined by real-time RT-PCR. Data are expressed as the mean ± SD. ### *p* < 0.001 versus the Control group. ** *p* < 0.01, *** *p* < 0.001 versus the Vehicle group. Con, control; Veh, vehicle; LE_R, reflux ethanol extract of *L. erythrorhizon*; DCS, N,N′-dicoumaroylspermidine; SP, spermidine.

Next, to examine whether N,N′-dicoumaroylspermidine has antiallergic effects on mast cells, we measured the amount of β-hexosaminidase in RBL-2H3 cells. RBL-2H3 cells were activated with an IgE-antigen complex, followed by the LE_R; the LE_R blocked the release of β-hexosaminidase. Additionally, we found that pretreating cells with N,N′-dicoumaroylspermidine inhibited degranulation under these conditions in a dose-dependent manner (Figure 8A). By contrast, spermidine had no effect on mast cell degranulation. Alleviation of allergy symptoms is due mostly to the manipulation of proinflammatory cytokines secreted by mast cells, and IL-3, IL-4, and IL-13 are regarded as critical therapeutic targets in allergic inflammatory illnesses [28]. Here, we found that N,N′-dicoumaroylspermidine reduced the release of IL-3, IL-4, and IL-13 by mast cells stimulated with an IgE–antigen complex (Figure 8B–D). These findings indicate that N,N′-dicoumaroylspermidine suppresses mast cell degranulation and proinflammatory cytokine production. 

### 2.7. Effects of N,N′-dicoumaroylspermidine on Serum Levels of OVA-Specific IgE and Inflammatory Cells in the NALF of OVA-Induced Allergic Rhinitis Mice Model

**Figure 9:**
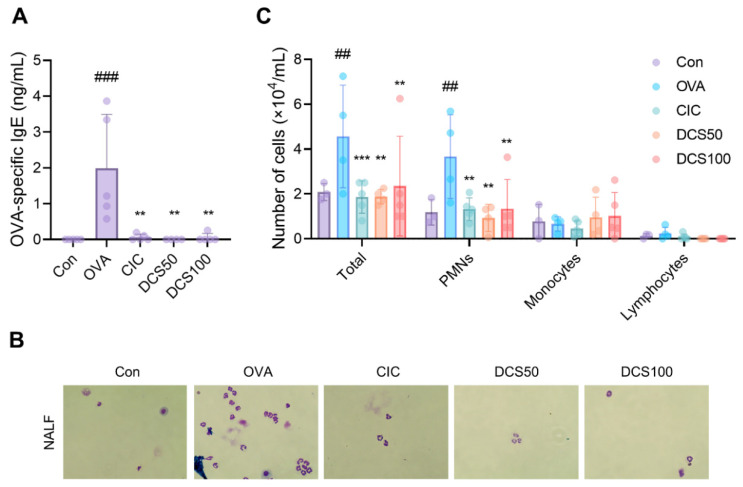
N,N′-dicoumaroylspermidine reduced the amount of OVA-specific IgE in serum, as well as infiltration of NAL by polymorphonuclear leukocytes. Mice in the CIC, DCS50, and DCS100 groups were sensitized and challenged with OVA prior to nasal administration of 15 μg of CIC, 50 μg of DCS, and 100 μg of DCS, respectively. (**A**) The amount of OVA-specific IgE in serum was measured in an ELISA. (**B**) Cytospin cell preparations were obtained after staining NALF with Diff-Quik (×400). (**C**) Total and individual cell numbers were counted using a hemocytometer. Data are expressed as the mean ± SD (*n* = 5 per group). ## *p* < 0.01, ### *p* < 0.001 versus the control group. ** *p* < 0.01, *** *p* < 0.001 versus the ovalbumin group. Con, control; OVA, ovalbumin; CIC, ciclesonide; DCS, N,N′-dicoumaroylspermidine; PMNs, polymorphonuclear leukocytes.

To further understand the antiallergic effects of N,N′-dicoumaroylspermidine, we used a mouse model of allergic rhinitis. Mice received daily intranasal N,N′-dicoumaroylspermidine for 7 days, followed by an intranasal challenge with OVA. We then measured the levels of OVA-specific immunoglobulins in the serum. The OVA group had significantly higher levels of OVA-specific IgE than the control group. However, upon intranasal treatment with CIC or N,N′-dicoumaroylspermidine (50 uM and 100 uM), serum OVA-specific IgE production fell markedly (Figure 9A). 

Next, we determined the leukocyte count in NALF. We counted the total number of cells, and the number of polymorphonuclear leukocytes (PMNs), monocytes, and lymphocytes. The OVA group had considerably more total cells than the control group, with the majority of these cells being PMNs. However, the cell counts in the N,N′-dicoumaroylspermidine (50 uM and 100 uM) and CIC groups (Figure 9B,C) were markedly lower. These results suggest that N,N′-dicoumaroylspermidine, an antiallergic component of *L. erythrorhizon*, suppresses OVA-induced allergic rhinitis by reducing OVA-specific IgE levels and infiltration by inflammatory cells.

## 3. Discussion

Despite the fact that allergic rhinitis is not a life-threatening condition, it has a significant impact on daily life and can incur substantial expenses. A sizable proportion of allergic rhinitis patients surveyed reported a diminished quality of life and sexual function, low self-esteem, and emotional exhaustion [29]. Traditional medicine has utilized plants for a very long time, and their chemical components are a significant source of new drug candidates for a variety of diseases. Since drugs derived from plants have few side effects in humans, it is prudent to identify plant components that can be used to treat allergic rhinitis [30]. In oriental medicine, the dried root of *L. erythrorhizon* is used primarily to increase blood flow, as an antipyretic, and as a detoxifier [19].

An allergic rhinitis animal model of producing an allergic response with OVA demonstrates nasal allergy symptoms comparable to those reported in humans, including Th2-related cytokine and chemokine secretion, the stimulation of Th2-type immune responses, and allergic behaviors including increased sneezing and nose rubbing [31,32]. After Almansouri et al., who reported an OVA-induced allergic rhinitis mouse model, this mouse model has been used in numerous experiments [33]. The goal of this study was to assess the antiallergic characteristics of an *L. erythrorhizon* reflux extract and its constituent compounds using OVA-induced allergic rhinitis and mast cell degranulation.

In this study, we identified 22 compounds in the hot reflux and/or cold extracts of *L. erythrorhizon*, including two new compounds, erythrin A and erythrin B, as well as N,N′-dicoumaroylspermidine, which is the first compound ever to be isolated from *L. erythrorhizon*. We found that an *L. erythrorhizon* reflux ethanol extract suppressed the allergic response in an OVA-induced allergic rhinitis mice model. We found here that N,N′-dicoumaroylspermidine did not affect the activity of NF-kB/AP1 and IRF, although it blocked mast cell degranulation and production of IL-3, IL-4, and IL-13. In addition, N,N′-dicoumaroylspermidine reduced the OVA-induced production of OVA-specific IgE and inflammatory cell infiltration. To the best of our knowledge, these are the first experimental data supporting the antiallergic rhinitis effects of N,N′-dicoumaroylspermidine extracted from *L. erythrorhizon*.

We analyzed and compared the chemical components of *L. erythrorhizon* obtained by hot reflux and cold extraction procedures. Since shikonin and shikonin derivatives are heat-labile, the HPLC peaks of extracts derived from the cold approach and the reflux method vary significantly [16,17]. Large quantities of shikonin and shikonin derivatives, including deoxyshikonin and isobutyrylshikonin, are destroyed by the hot reflux method [17]. Under hot conditions, in which shikonin and its derivatives are essentially absent, we were able to analyze a number of substances. Erythrin A and erythrin B were found to have a very similar structure. After NMR analysis, we proposed that they likely comprise two isomers. 

Mast cells are immune cells that play a significant part in the development of allergic reactions [34]. They are situated on body surfaces, such as the skin, respiratory tract, and gastrointestinal tract, which are in contact with the external environment and are suitable sites on which to react to pollen and pathogens [35]. After secondary allergen exposure, mast cells primarily increase IgE-mediated allergy responses. Through the activation of FcεRI, a high-affinity IgE receptor, mast cells mediate acute and chronic inflammation [34]. The rat basophilic leukemia cell line RBL-2H3 expresses a significant amount of FcεRI, making it a good choice for studies involving IgE-mediated mast cell activation [36]. We investigated the antiallergic action of N,N′-dicoumaroylspermidine in RBL-2H3 cells in vitro and found that it suppressed the release of β-hexosaminidase, a degranulation marker, as well as the secretion of IL-3, IL-4, and IL-13.

IL-4 and IL-13 are produced by activated T cells, B cells, eosinophils, and mast cells, and are classified as Th2 cytokines [37]. IL-4 transforms the Ig class to IgE, enhances the production of adhesion molecules, and stimulates eosinophil invasion into the inflammatory region in order to provoke an allergic response [37]. IL-13 promotes B cell maturation, differentiation, and IgE isotype conversion [38]. The reflux ethanol extract of *L. erythrorhizon* significantly decreased the levels of IL-4 and IL-13 in NALF, whereas N,N′-dicoumaroylspermidine suppressed the expression of IL-4 and IL-13 in mast cells. Therefore, we suggest that N,N′-dicoumaroylspermidine, one of the chemical constituents of *L. erythrorhizon*, has antiallergic effects on allergic rhinitis through influencing Th2 cytokines.

N,N′-dicoumaroylspermidine is a plant secondary metabolite found in *Helianthus annuus* (sunflower), *Brassica alboglabra Bailey* (Chinese kale), *Vicia faba* (fava bean), *Oryza sativa* (rice), and *Pyrus communis* (pear) [39,40,41,42]. Structurally, it is a conjugate of phenolamide, coumaric acid, and spermidine. According to one report, when the white-backed planthopper (an insect herbivore) invades rice, the rice produces 11 types of phenolamide, including N,N′-dicoumaroylspermidine [40]. It is thought that N,N′-dicoumaroylspermidine plays a role in plant defense similar to that of phenolamide, which is induced by pathogen infection, herbivore infection, and UV radiation [43,44,45,46]. However, no study has examined the biological activity of isolated N,N′-dicoumaroylspermidine. Here, we isolated N,N′-dicoumaroylspermidine from *L. erythrorhizon* and revealed for the first time that it has an antiallergy effect against allergic rhinitis both in vitro and in vivo. N,N′-dicoumaroylspermidine was the only compound isolated from *L. erythrorhizon* that did not impair the viability of macrophages and mast cells. Although N,N′-dicoumaroylspermidine did not suppress the activation of NF-kB/AP-1 and IRF, it did prevent IgE-mediated mast cell degranulation and the secretion of IL-3, IL-4, and IL-13. In the OVA-induced allergic rhinitis mouse model, N,N′-dicoumaroylspermidine decreased the OVA-specific IgE levels in the serum and reduced PMN infiltration into NALF.

Since *p*-coumaric acid has antiallergic activity in a β-hexosaminidase release assay, it is possible that the bioiological activity of N,N′-dicoumaroylspermidine may be mediated by *p*-coumaric acid [47]. Based on our finding that spermidine, the *p*-coumaric acid-free form of N,N′-dicoumaroylspermidine, does not prevent the release of β-hexosaminidase, we deduce that *p*-coumaric acid in N,N′-dicoumaroylspermidine plays a crucial role in its antiallergic activity.

## 4. Materials and Methods

### 4.1. Plant Material and Extraction

Roots of *L. erythrorhizon* were purchased from Kyungdong market (Seoul, Korea), and a voucher specimen (KIST-053) was deposited at the herbarium of the KIST Gangneung Institute. The reflux and cold methods, both using 95% ethanol, were used to obtain two extracts. For reflux extraction, 10 kg of dried roots, which were ground by a large scale crusher into small pieces of 0.5–1 cm, were extracted three times (for 3 h each) at 70 °C. The residue (reflux extract) was obtained after removing the solvent under reduced pressure. For cold extraction, 2 kg of dried roots was macerated in 95% ethanol for 24 h (3 times) at room temperature, followed by removal of all ethanol under vacuum to yield the residue (cold extract). Both extracts were kept at −20 °C before using in the experiments.

### 4.2. HPLC Analysis

Analyses were conducted using an Agilent (Palo Alto, CA, USA) series 1200 liquid chromatograph and a YMC hydrosphere C_18_ column packed with 5 µm particles (4.6 × 250 mm) maintained in a 25 °C column oven. The running method is described in Table S1. Formic acid (0.1%) in both acetonitrile and water was used as a mobile phase to achieve a better peak shape. The injection volume was 10 µL, the flow rate was 0.7 mL/min, and UV detection was performed at 280 nm for fingerprinting.

### 4.3. Isolation and Identification

The reflux extract was suspended in water and then partitioned with n-hexane, dichloromethane, ethyl acetate, and n-butyl alcohol. The hexane-soluble fraction was applied to the RP-18 column along with MeOH to yield three subfractions (Hx 1–3). Subfraction Hx-1 was then applied to a Sephadex LH-20 open column and eluted with MeOH-H_2_O (1:5 to 10:1) to yield compound 3 (2.1 mg) and compound 13 (3.1 mg). Subfraction Hx-1 was loaded to the RP-18 column along with MeOH to obtain two new isomer compounds, 1 and 2, as a mixture (2.0 mg). From the MC-soluble fraction, compounds 14 (3.4 mg), 15 (2.1 mg), 16 (1.9 mg), and 17 (2.5 mg) were collected after elution from a silica gel column and then purified over Sephadex LH-20. Similarly, compounds 19 (3.4 mg), 20 (3.4 mg), 21 (3.4 mg), and 22 (3.4 mg) were obtained from the EA-soluble fraction after isolation by repeated elution from a Sephadex LH-20 open column using MeOH-H_2_O (2:10 to 10:1).

Different from the reflux extract, the cold extract was applied directly to a silica gel column in a stepwise gradient of n-hexan-EtOAc (10:1 to 0:1) to yield compounds **5** (3.2 mg) and **9** (4.8 mg), and two other fractions (1 and 2). These fractions were loaded onto an RP-18 column and further purified over Sephadex LH-20 using MeOH-H_2_O (1:5 to 10:1) to yield compounds **6** (5.4 mg), **8** (6.1 mg), and **18** (2.1 mg). Using the same isolation method, compounds **4** (4.2 mg), **7** (3.5 mg), **10** (2.8 mg), **11** (4.6 mg), and **12** (2.3 mg) were isolated from fraction 2.

The mixture of erythrin A (**1**) and erythrin B (**2**): an orange gum; UV λ_max_ (ε) (chloroform): 210 (154), 280 (45), 540 (48); ^1^H-NMR and ^13^C-NMR (CHCl_3_-*d*) data; see Table 1, Figures S1–S6; HR-ESIMS *m*/*z*: 393.1339 [M-H]^−^ (calcd. for C_36_H_29_O_16_ 393.1338). 

N^1^‴,N^3^‴-dicoumaroylspermidine (**3**): a colorless gum; UV λ_max_ (ε) (methanol): 210 (380), 300 (590); ^1^H-NMR and ^13^C-NMR (CH_3_OH-*d*_4_) data, see Table 2, Figure S7–S12; HR-ESIMS *m*/*z*: 438.2387 [M+H]^+^ (calcd. for C_25_H_32_N_3_O_4_ 438.2393).

### 4.4. Chemicals and Apparatus

The 1D and 2D NMR spectra were obtained using a Bruker Avance DRX Spectrometer, and the chemical shifts were recorded as δ values (ppm). Mass spectra were recorded using a high-resolution ESI Mass spectrometer. Silica gel (Merck, 63–200 μm particle size) and RP-18 (Merck, 75 μm particle size) were used for column chromatography. TLC was performed using Merck silicagel 60 F254 and RP-18 F254 plates. Isolated compounds were visualized after spraying with aqueous 20% H_2_SO_4_ and heating for about 5 min. Analytical-grade acetonitrile and distilled HPLC-grade water were purchased from Fisher Scientific (Pittsburgh, PA, USA). Open column chromatography was conducted using silica gel (Merck, Darmstadt, Germany) and Sephadex LH-20 (Pharmacia, Uppsala, Sweden).

### 4.5. Cell Culture and Viability Assay

The RBL-2H3 cell line was bought from the Korean cell line bank’s Korean cell line research foundation (Seoul, Korea) and grown in culture dishes in DMEM supplemented with 10% fetal bovine serum (FBS) and 100 U/mL penicillin/streptomycin. Cultures were kept at 37 °C in a humidified environment containing 5% CO_2_. THP-1 Xblue and THP-1 Blue ISG cells were purchased from Invivogen (Invivogen, San Diego, CA, USA). THP-1 Xblue and THP-1 Blue ISG cells were cultured in RPMI supplemented with 10% FBS, 100 U/mL penicillin/streptomycin, and 200 μg/mL zeocin (InvivoGen, San Diego, CA, USA). Cultures were kept at 37 °C in a humidified environment containing 5% CO_2_. MTT (3-(4,5-dimethylthiazol-2-yl)2,5-diphenyltetrazolium bromide) solution was applied to the cells (final concentration: 0.5 mg/mL) for 1 h at 37 °C to determine cell viability. Both extracts of *L. erythrorhizon* and the indicated compounds were dissolved in dimethyl sulfoxide (DMSO) and diluted with culture medium when treated with cells. A spectrophotometer (BioTek Instruments, Winooski, USA) with a 570 nm test wavelength and a 690 nm reference wavelength was used to evaluate the optical density of the solubilized formazan product.

### 4.6. β-Hexosaminidase Assay

The RBL-2H3 cells (5 × 10^5^ cells/well) were seeded on a 24-well plate and activated with DNP-IgE (50 ng/mL) for 24 h. Then, the cells were treated for 1 h with LE_R and N,N′-dicoumaroylspermidine, followed by stimulation with DNP-BSA (100 ng/mL) in Siraganian buffer (119 mM NaCl, 5.6 mM Glucose, 0.4 mM MgCl_2_, 0.1 percent BSA, 5 mM KCL, 25 mM PIPES, 1 mM CaCl_2_, pH 7.2). The color change in the substrate (1 mM *p*-nitrophenyl-N-acetyl—d-glucosaminide) in citrate buffer was used as a readout for β-hexoaminidase activity. 

### 4.7. Real-Time Quantitative PCR

The RNeasy Mini kit (QIAGEN, Hilden, Germany) was used to extract total RNA from collected RBL-2H3 cells. SuperScript III Reverse Transcriptase (Invitrogen, Carlsbad, CA, USA) was used to synthesize cDNA. The TaqMan Gene Expression Assay, TaqMan Fast advanced Master Mix, and QuantStudio 6 were then used to amplify cDNA (Thermo Fisher Scientific, Waltham, MA, USA). The 2^−ΔΔCT^ comparative method was used for data analysis and calculations, as per the manufacturer’s instructions.

### 4.8. Measurement of NF-κB/AP-1 and IRF Activity

THP-1 XBlue and THP-1 Blue ISG cells (1 × 10^5^ cells/well) were seeded into a 96-well plate, treated with N,N′-dicoumaroylspermidine dissolved in DMSO for 1 h, and then stimulated for 24 h at 37 °C with Pam3CSK4 (100 ng/mL), zymosan A (10 μg/mL), or LPS (100 ng/mL). After incubation of supernatants for 24 h with Quanti-Blue medium (InvivoGen, San Diego, CA, USA), the reaction was incubated for 30 min, and then the absorbance was measured at 630 nm on the multiplate reader (BioTek Instruments, Winooski, VT, USA).

### 4.9. In Vivo Studies of OVA-Induced Allergic Rhinitis Mouse Model

Six-week-old female BALB/c mice (Orient Bio Inc., Seong-nam, Korea) were kept in an air-conditioned room with a 12 h light/dark cycle, a temperature of 23 ± 2 °C, and a relative humidity of 55 ± 10%. All experimental protocols were approved by the KIST animal care and use committee (approval NO. 2020-155).

For the OVA-induced allergic rhinitis mouse model, four groups of BALB/c mice (*n* = 10) were assigned randomly to (1) a control (Con) group, (2) an OVA group, (3) an LE_R group, and (4) a CIC group. Mice were sensitized three times by intraperitoneal injection of 100 μg of ovalbumin (OVA; Grade V, Sigma-Aldrich, St. Louis, MO, USA) and 2 mg of aluminum hydroxide (Sigma-Aldrich) on Days 0, 7, and 14. From Days 21 to 27, sensitized mice were challenged by intranasal delivery of 500 μg of OVA (20 μL into each nasal cavity). Mice in the LE_R groups were given 50 mg/kg of LE_R (once a day by oral gavage, 100 μL) after being sensitized and challenged with OVA. Mice in the CIC group were sensitized and challenged with OVA before receiving 15 μg of CIC intranasally. Mice in the DCS group were sensitized and challenged with OVA before receiving 50 μg or 100 μg of N,N′-dicoumaroylspermidine intranasally. Con mice were not sensitized, challenged, or manipulated in any way. On Day 28, mice were sedated with ether and sacrificed.

### 4.10. Collection and Analysis of Serum and NALF

One day after the final intranasal challenge, blood samples were taken from the abdominal caval vein. After collection of the whole blood, the blood was left undisturbed at room temperature. The whole blood removed clots by centrifugation at 1000~5000× *g* for 10 min. The supernatant was collected and kept at −80 °C until needed for cytokine measurements. NALF was collected by introducing a tube into the upper section of the trachea in the nasal direction and rinsing with 500 mL of saline. The supernatant was collected and kept at −80 °C until needed for cytokine measurements. Real-time quantitative PCR and the ELISA MAXTM Deluxe Set KIT (BioLegend, San Diego, CA, USA) were used to examine the amounts of IL-4, IL-5, and IL-13. Total cells and individual cell types in NALF were counted using a standard procedure based on Diff-Quik staining (Siemens, Newark, DE, USA); cells from all three lavages were pooled.

### 4.11. Histopathological Evaluation of Nasal Mucosa

Head tissue slides were stained with H&E using a standard procedure. The slides were treated with hematoxylin buffer at room temperature, rinsed three times with distilled water, and immersed in a 1% eosin Y solution. A PAS stain kit was used to stain the slides with PAS (ab150680; Abcam, Cambridge, UK). A conventional method was used to stain slides with toluidine blue. The slides were treated with toluidine blue working solution for 3 min. The slides were then washed three times with distilled water before being mounted and examined under a CKX41 inverted phase contrast microscope (Olympus, Tokyo, Japan).

### 4.12. Statistical Analysis

Statistical comparisons were made using a paired Student’s *t*-test or one-way ANOVA in GraphPad Prism 9.0 (GraphPad Software, San Diego, CA, USA). Values are expressed as the mean ± standard deviation (SD) of independent experiments. 

## 5. Conclusions

In conclusion, an *L. erythrorhizon* reflux ethanol extract protected mice from the OVA-induced allergic rhinitis model. Among the 22 compounds identified in the *L. erythrorhizon* extracts, N,N′-dicoumaroylspermidine inhibited mast cell degranulation and cytokine releases in vitro, and prevented OVA-induced allergic rhinitis responses in vivo. Thus, our findings suggest that N,N′-dicoumaroylspermidine isolated from *L. erythrorhizon* has the potential to improve allergic rhinitis symptoms.

## Figures and Tables

**Table 1:** ^13^C and ^1^H-NMR data for compound **1** and **2** (at 400 MHz for ^1^H-NMR and 100 MHz for ^13^C-NMR in CD_3_OD, ^δ^ in ppm, *J* in Hz).

Position	1		2	
	^δ^ C	^δ^ H	^δ^ C	^δ^ H
1	157.40, C		157.34, C	
2	125.29, CH	7.31, s	125.04, CH	7.30, s
3	142.36, C		142.11, C	
4	154.99, C		154.95, C	
5	127.39, CH	8.16, m	127.20, CH	8.16, m
6	145.97, C		145.89, C	
7	135.36, CH	7.65, m	135.43, CH	7.65, m
8	127.3, CH	8.26, d (7.96)	127.30, CH	8.24, d (7.96)
9	186.63, C		186.97, C	
10	187.18, C		187.33, C	
4a	112.68, C		112.52, C	
8a	131.21, C		131.20, C	
9a	112.23, C		112.08, C	
10a	133.41, C		133.38, C	
1′	70.09, CH	6.20, m	70.09, CH	6.20, m
2′	33.01, CH_2_	2.49–2.73, m	33.01, CH_2_	2.49–2.73, m
3′	118.13, CH	5.17, m	118.13, CH	5.17, m
4′	135.67, C		135.67, C	
5′	17.88, CH_3_	1.59, s	17.88, CH_3_	1.59, s
6′	25.81, CH_3_	1.71, s	25.81, CH_3_	1.71, s
7′	170.10, C		170.10, C	
8′	21.10, CH_3_	2.16, s	21.10, CH_3_	2.16, s
9′	22.03, CH_3_	2.56, s	22.03, CH_3_	2.56, s
OH-1		12.98		12.94
OH-4		13.39		13.43

**Table 2:** ^13^C and ^1^H-NMR data for compound **3** (at 400 MHz for ^1^H-NMR and 100 MHz for ^13^C-NMR in CD_3_OD, ^δ^ in ppm, *J* in Hz).

Position	3	
	^δ^ C	^δ^ H
1	140.73, CH	7.51, d (15.56)
2	116.24, CH	6.42, d (15.68)
3	168.93, C	
4	35.56, CH_2_	3.43, t (6.40)
5	26.58, CH_2_	1.89–1.99, m
6	44.96, CH_2_	2.99–3.08, m
7	47.62, CH_2_	2.99–3.08, m
8	26.32, CH_2_	1.65–1.82, m
9	23.28, CH_2_	1.65–1.82, m
10	38.10, CH_2_	3.37, t (6.72)
11	168.05, C	
12	116.91, CH	6.42, d (15.68)
13	141.40, CH	7.47, d (15.56)
1′	126.02, C	
2′	129.17, CH	7.41, d (8.64)
3′	115.36, CH	6.80, d (8.56)
4′	159.26, C	
5′	115.36, CH	6.80, d (8.56)
6′	129.17, CH	7.41, d (8.64)
1″	126.18, C	
2″	129.30, CH	7.42, d (8.68)
3″	115.39, CH	6.80, d (8.56)
4″	159.42, C	
5″	115.39, CH	6.80, d (8.56)
6″	129.30, CH	7.42, d (8.68)

## Data Availability

The data presented in this study are available from the corresponding author upon reasonable request.

## Supplementary Materials

The following supporting information can be downloaded at: https://www.mdpi.com/article/10.3390/ijms231810403/s1.
