# Supplementary material for: Antiallergic Effects of N,N-dicoumaroylspermidine Isolated from Lithospermum erythrorhizon on Mast Cells and Ovalbumin-Induced Allergic Rhinitis
Source: Int J Mol Sci. 2022 Sep 8;23(18):10403. doi: 10.3390/ijms231810403 (PMC9499623; doi:10.3390/ijms231810403)
Supplement: Supplementary file 1 [file ijms-23-10403-s001.zip › ijms-1907587-supplementary.pdf]

Supplementary data

List of Figures

Figure S1. HR ESI-MS spectrum of the mixture of two new compound **1** and **2**

Figure S2. ^{1}H-NMR spectrum of the mixture of two new compound **1** and **2** in $CHCl_3$-*d* at 400 MHz

Figure S3. ^{13}C-NMR spectrum of the mixture of two new compound **1** and **2** in $CHCl_3$-*d* at 100 MHz

Figure S4. HSQC spectrum of the mixture of two new compound **1** and **2** in $CHCl_3$-*d*

Figure S5. COSY spectrum of the mixture of two new compound **1** and **2** in $CHCl_3$-*d*

Figure S6 .HMBC spectrum of the mixture of two new compound **1** and **2** in $CHCl_3$-*d*

Figure S7. HR ESI-MS spectrum of compound **3**

Figure S8. ^{1}H-NMR spectrum of compound **3** in CH_3OH-d_4 at 400 MHz

Figure S9. ^{13}C-NMR spectrum of compound **3** in CH_3OH-d_4 at 100 MHz

Figure S10. HSQC spectrum of compound **3** in CH_3OH-d_4

Figure S11. COSY spectrum of compound **3** in CH_3OH-d_4

Figure S12. HMBC spectrum of compound **3** in CH_3OH-d_4

Table S1. HPLC method

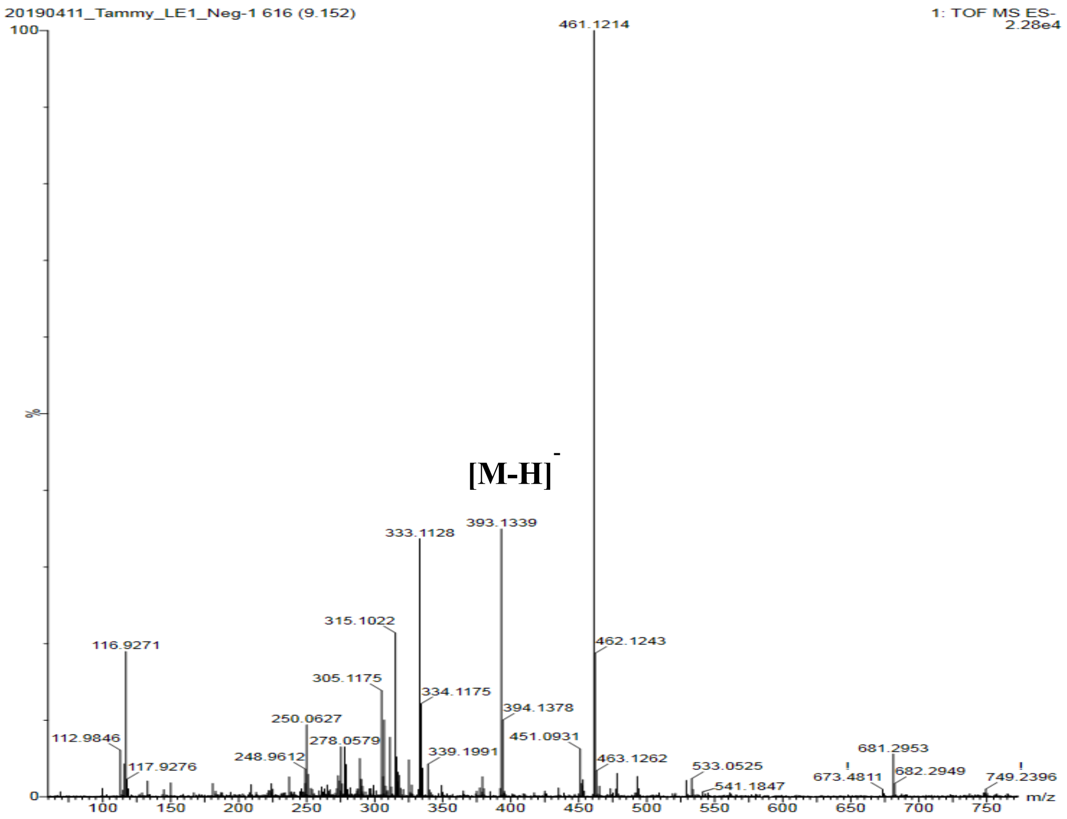

Figure S1. HR ESI-MS spectrum of the mixture of two new compound **1** and **2**

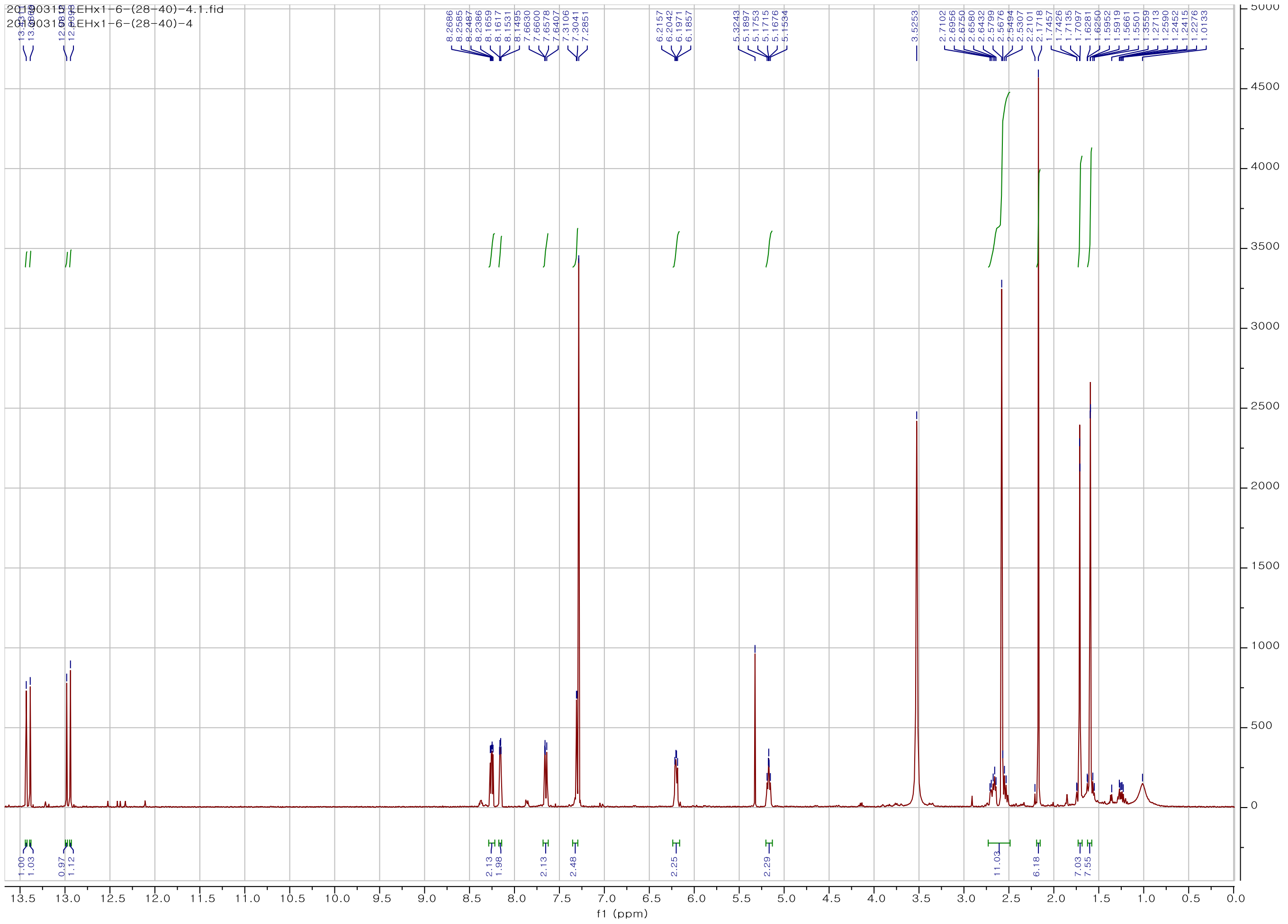

Figure S2. ^{1}H-NMR spectrum of the mixture of two new compound **1** and **2** in $CHCl_3$-*d* at 400 MHz

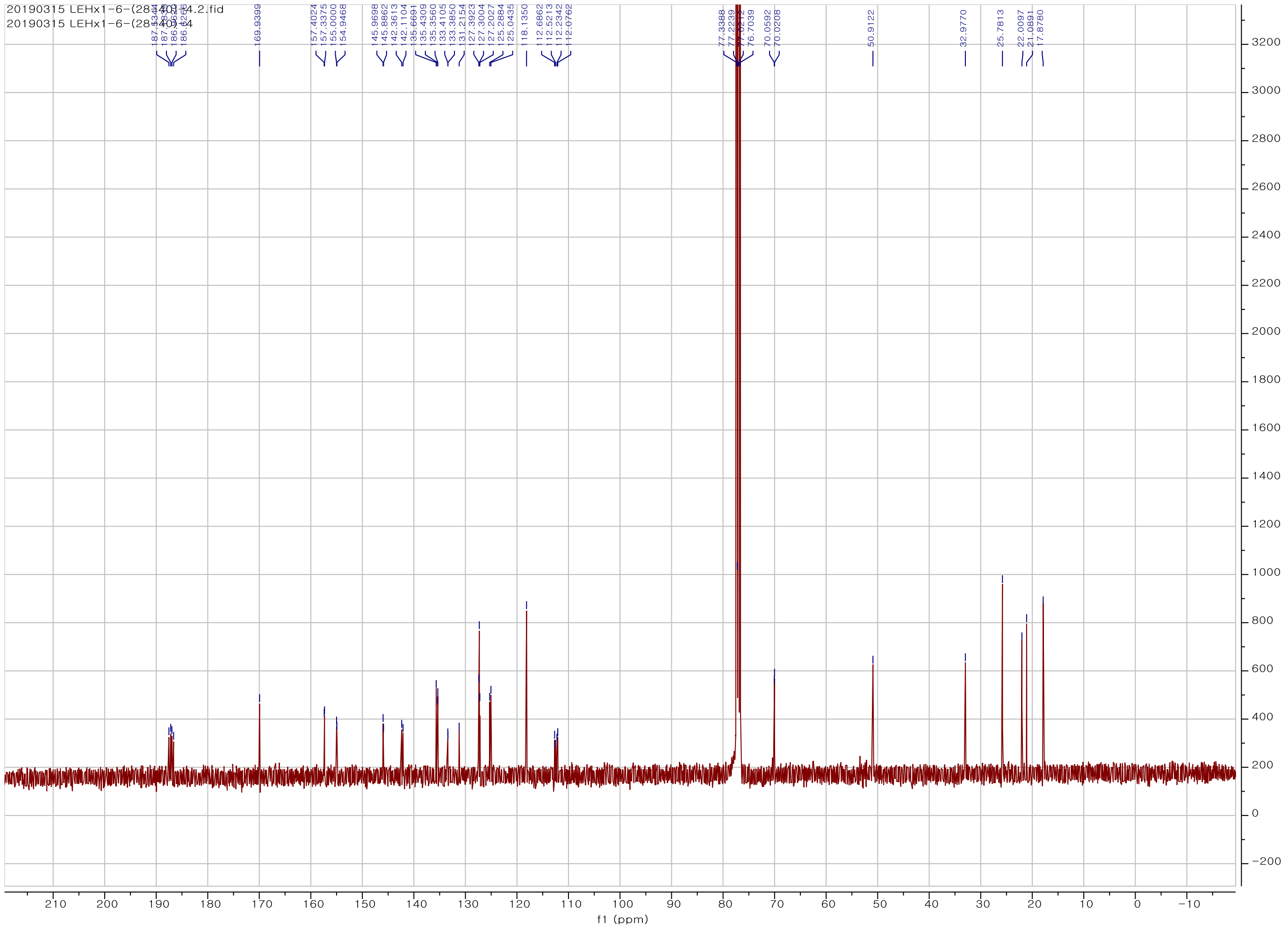

Figure S3. ^{13}C-NMR spectrum of the mixture of two new compound **1** and **2** in $CHCl_3$-*d* at 100 MHz

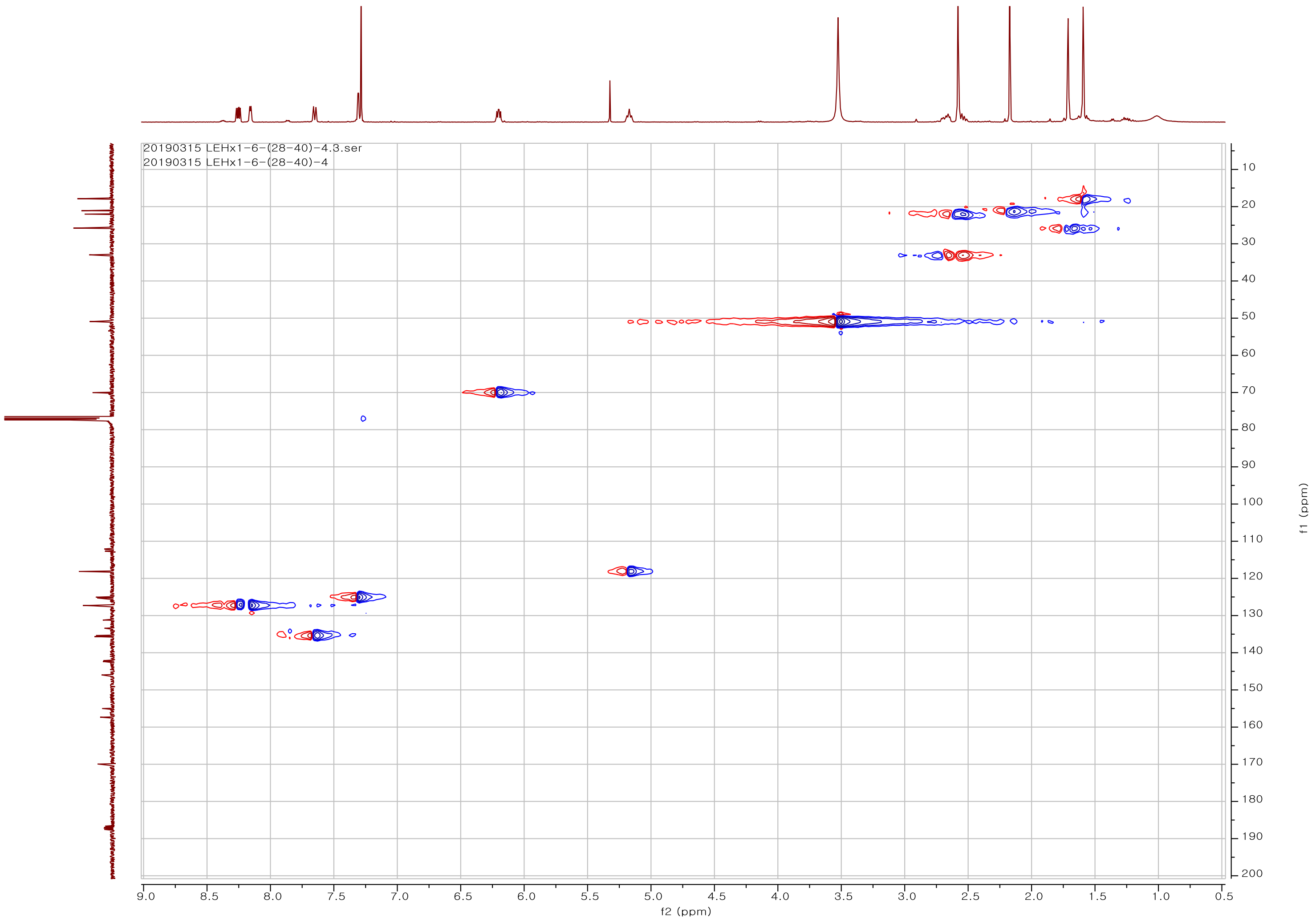

Figure S4. HSQC spectrum of the mixture of two new compound **1** and **2** in $CHCl_3$-*d*

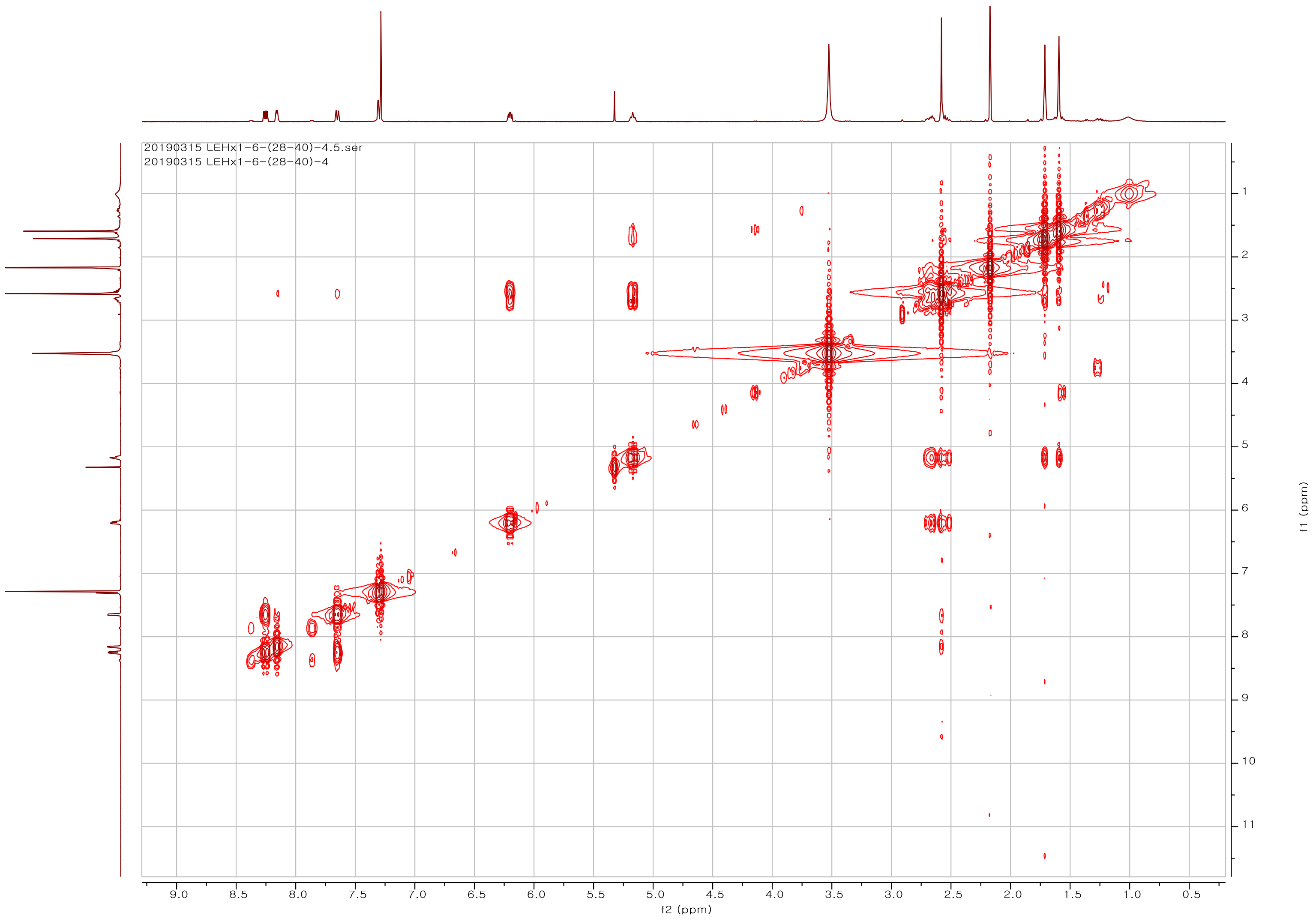

Figure S5. COSY spectrum of the mixture of two new compound **1** and **2** in $CHCl_3$-*d*

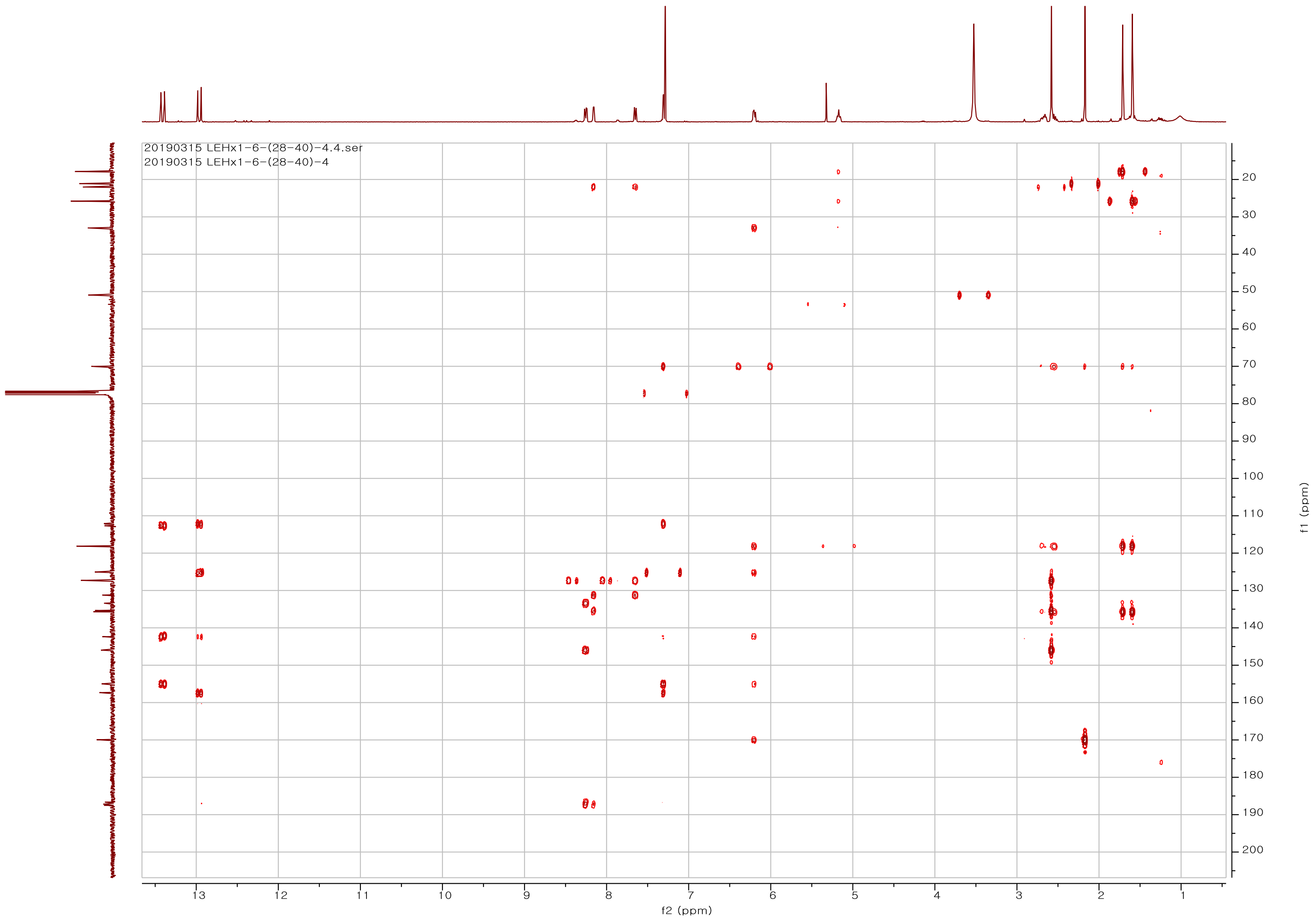

Figure S6. HMBC spectrum of the mixture of two new compound **1** and **2** in $CHCl_3$-*d*

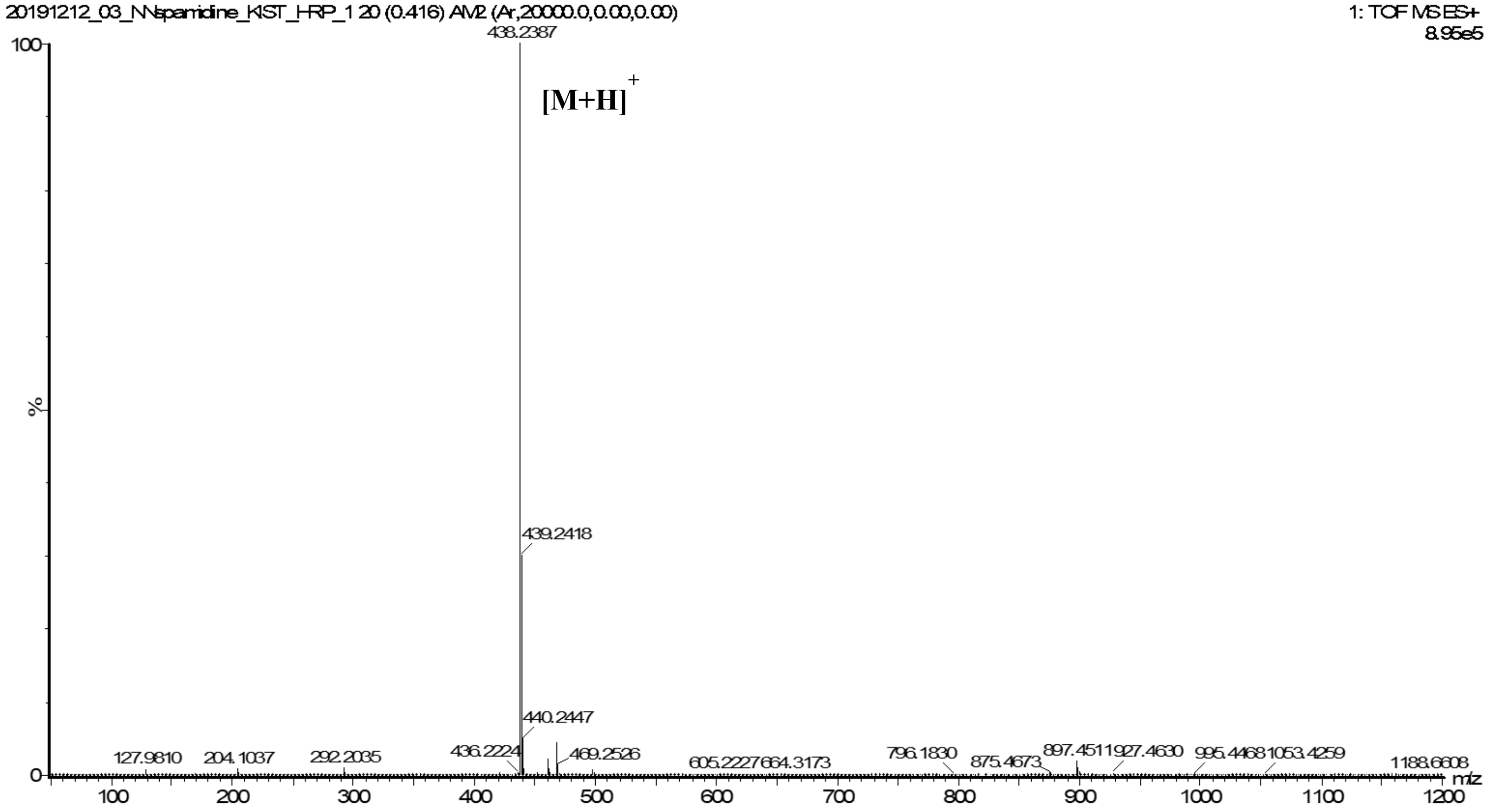

Figure S7. HR ESI-MS spectrum of compound **3**

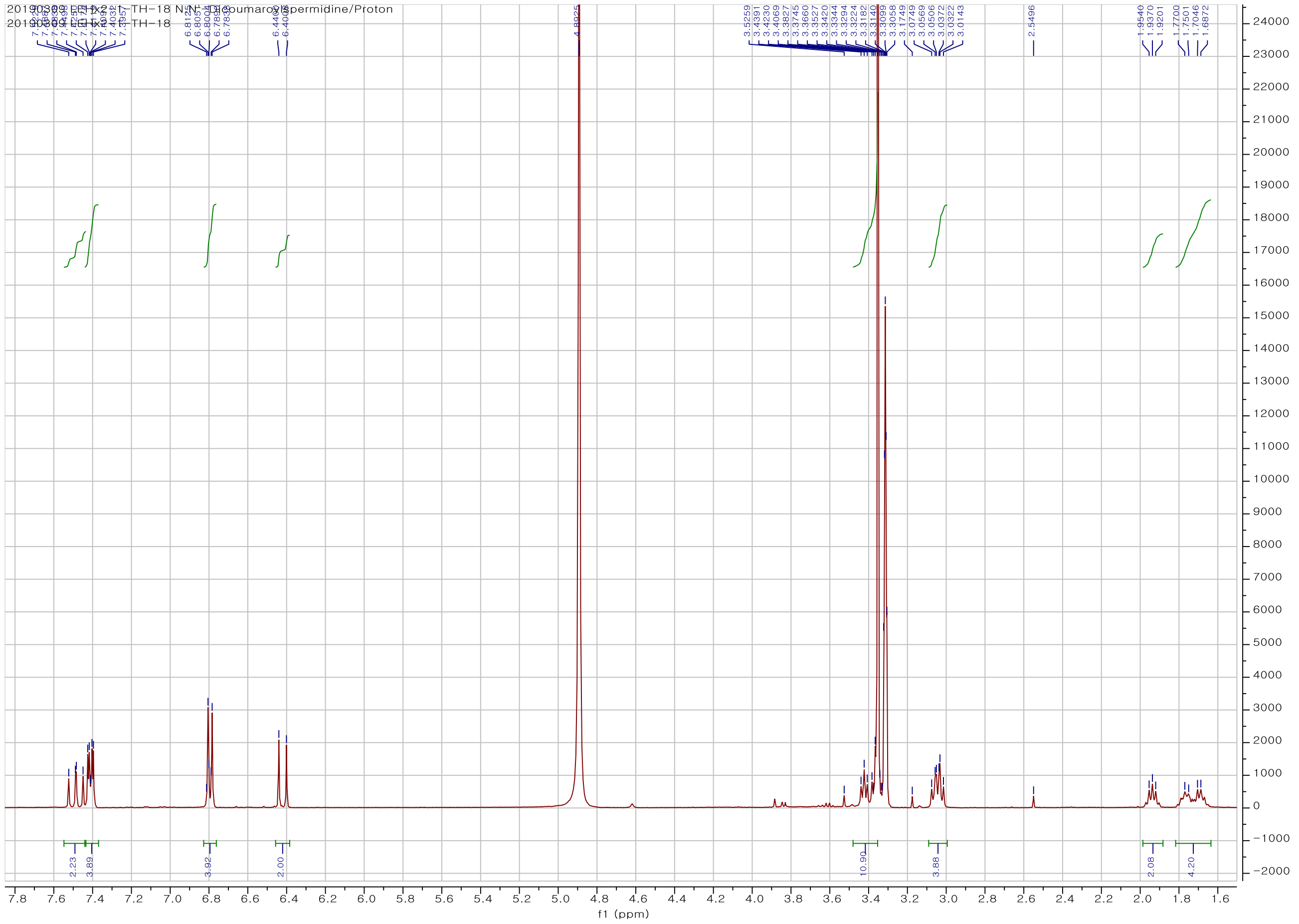

Figure S8. ^{1}H-NMR spectrum of compound **3** in CH_3OH-d_4 at 400 MHz

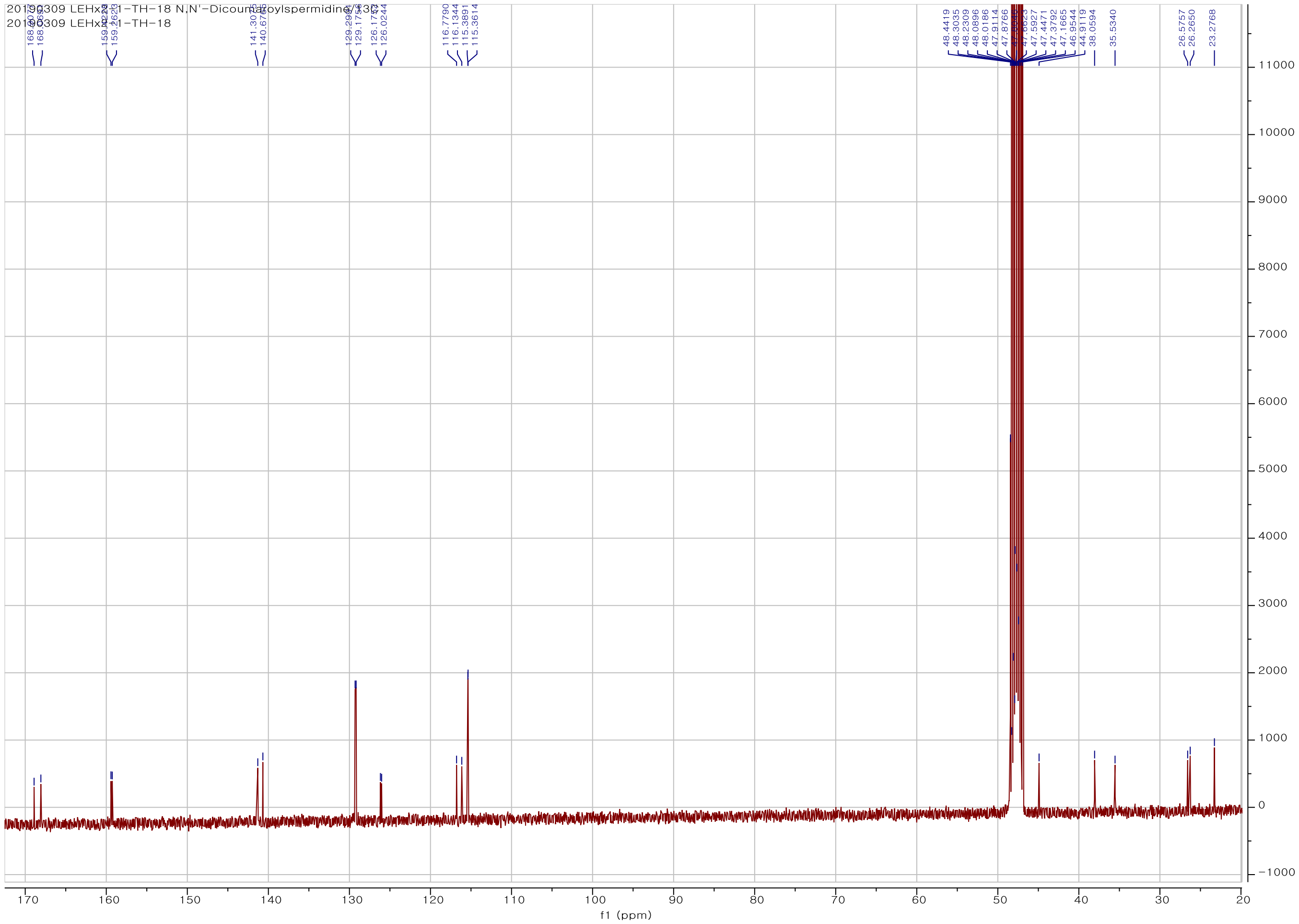

Figure S9. ^{13}C-NMR spectrum of compound **3** in CH_3OH-d_4 at 100 MHz

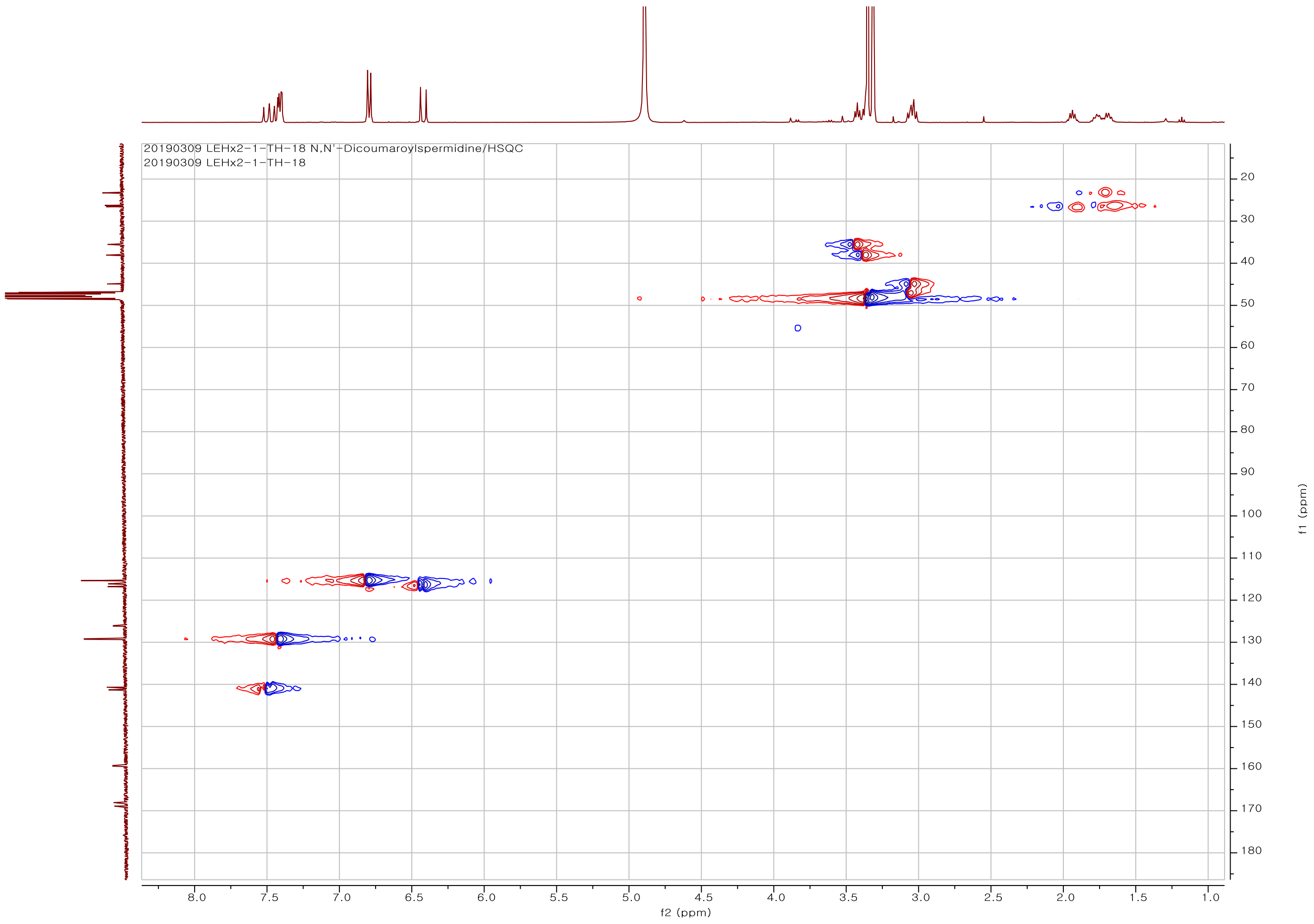

Figure S10. HSQC spectrum of compound **3** in CH_3OH-d_4

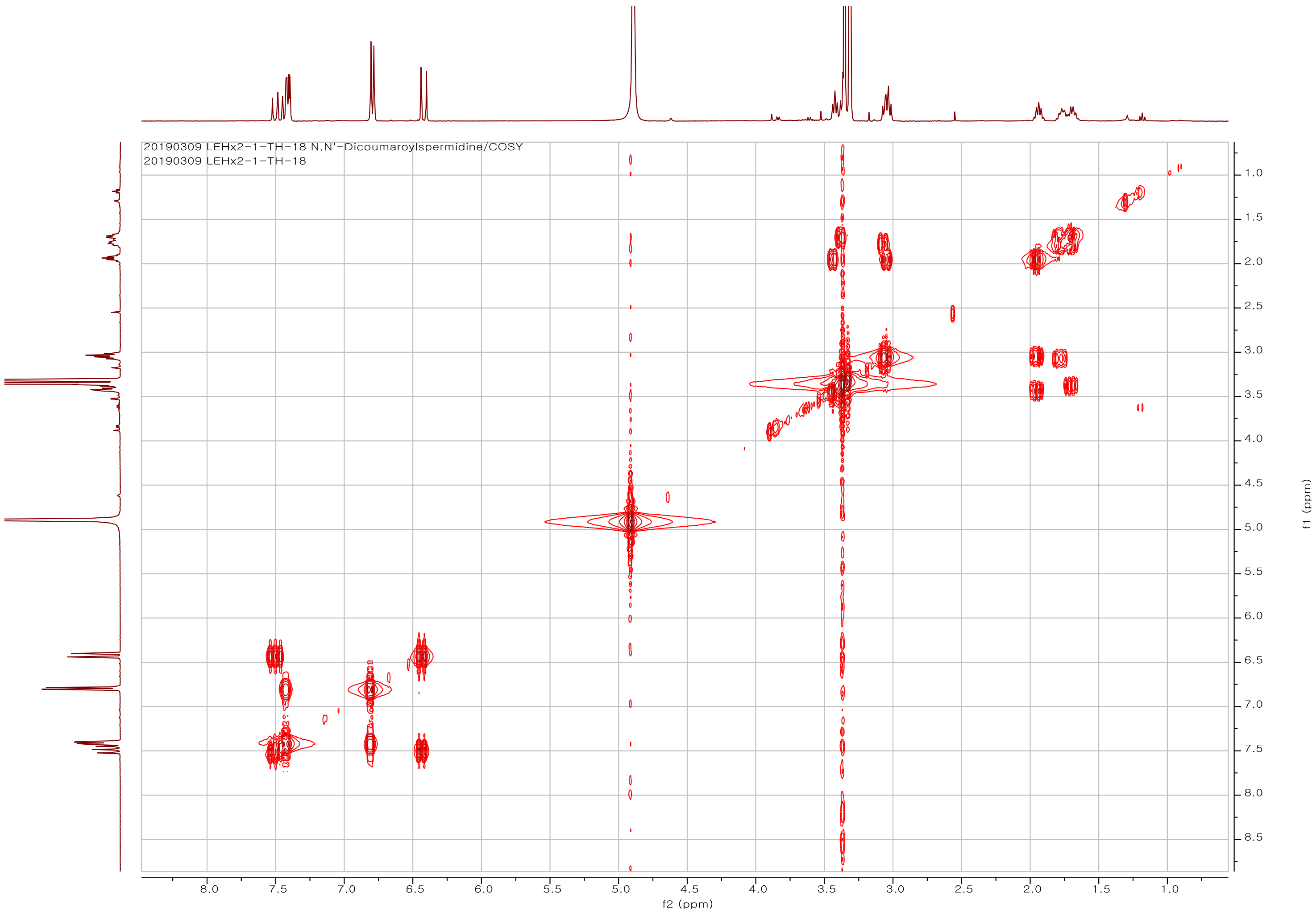

Figure S11. COSY spectrum of compound **3** in CH_3OH-*d*$_4$

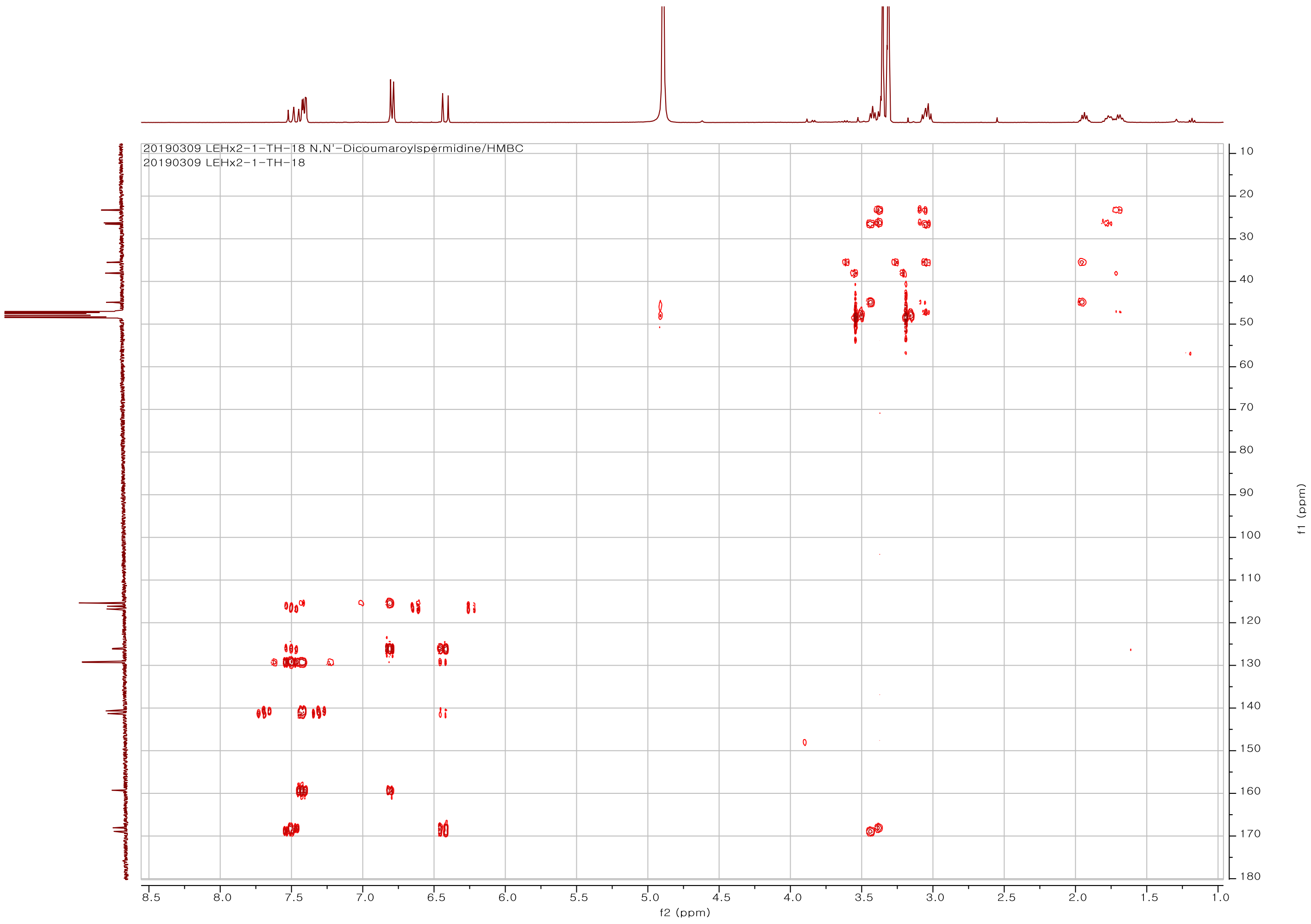

Figure S12. HMBC spectrum of compound **3** in CH_3OH-d_4

Time (min)	0.1% formic acid in water (%)	0.1% formic acid in acetonitrile (%)
0	95	5
2	95	5
40	50	50
60	50	50
61	30	70
71	30	70
72	20	80
85	20	80
90	5	95
95	95	5

Table S1. HPLC method
